# EHMTI-0120. Are the IHS’ recommendations for evaluation and registration of adverse events in drug trials in migraine being followed ?

**DOI:** 10.1186/1129-2377-15-S1-G37

**Published:** 2014-09-18

**Authors:** P Tfelt-Hansen, J Kauffmann

**Affiliations:** 1 Neurology, Danish Headache Center, Glostrup, Denmark; 2 Neurology, North zealand Hospital, Hillerød, Denmark

## Introduction

In 2008 the International Headache Society (IHS) published guidelines on “evaluation and registration of adverse events in clinical drug trials in migraine (Cephalalgia 2008; 28: 683-688). In this study we evaluated whether these guidelines on adverse events (AEs) subsequently were adhered to by reviewing , randomized, controlled trials (RCTs) on migraine drug treatment published in the three leading headache journals for the last 4 years.

## Methods

We reviewed all double-blind RCTs on acute and preventive migraine drugs appearing in 2010, 2011, 2012, and 2013 in Headache, Journal of Headache and Pain, and Cephalalgia.

We noted for each RTC the presentations of 5 parameters: number of patients with any AEs, any serious AEs, patients withdrawn because of AEs, intensity of AEs, see results.

## Results

At total of 23 RCTs , 17 acute treatment RCTs and 6 preventive RCTs, were reviewed.

Patients with any AEs were reported in 16 of 23 RCTs; any serious AEs were reported in 8 of 23 RCTs; patients withdrawn because of AEs were reported in 5 of 23 RCTs; and intensity of AES was mentioned in 8 of 23 RCTs (in most cases e.g. most AEs were mild and moderate).

## Discussion

It is noteworthy that even such an obvious major parameter for tolerability, patients with any AES, was only reported in 70% (95% CI: 51 – 89%) of the reviewed RCTs. In addition, intensity of AEs is only very briefly reported.

No conflict of interest.

